# Psychometric properties of Persian version of individual innovativeness scale among nursing students: a cross-sectional study

**DOI:** 10.1186/s12909-023-04641-2

**Published:** 2023-09-14

**Authors:** Naval Heydari, Mahnaz Rakhshan, Camellia Torabizadeh, Ghasem Salimi

**Affiliations:** 1 Student Research Committee, Department of Nursing, School of Nursing and Midwifery, Shiraz University of Medical Sciences, Shiraz, Iran; 2 Community Based Psychiatric Care Research Center, Department of Nursing, School of Nursing and Midwifery, Shiraz University of Medical Sciences, Shiraz, Iran; 3 Department of Educational Administration and Planning, Faculty of Education and Psychology, Shiraz University, Shiraz, Iran

**Keywords:** Innovation, Innovativeness, Students, Nursing, Questionnaire

## Abstract

**Background:**

One of the basic necessities for fostering innovation in nursing students is to study the level of individual innovation using an appropriate tool. This study was conducted with the aim of translation and psychometric analysis of 20-item individual innovativeness scale (IIS) developed by Hurt et al., among Iranian nursing students.

**Methods:**

This cross-sectional study was performed on 140 nursing students between September 2020 and June 2021 in one of the southern cities of Iran. IIS was translated through forward-backward method, and its face validity and content validity were examined quantitatively and qualitatively. Then, its construct validity was measured by exploratory factor analysis, and its stability and internal consistency were examined.

**Results:**

The evidence of qualitative face validity and content validity of IIS were observed. The impact score was higher than 1.5, content validity ratio was between 0.6 and 1, content validity index was between 0.8 and 1, and SCVI-Average was 0.91. Based on exploratory factor analysis, three sub-scales were extracted that explained 55.49% of the changes in the questions. Cronbach’s alpha and intraclass correlation coefficient were 0.880 and 0.949, respectively.

**Conclusion:**

The Persian version of IIS had acceptable validity and reliability. Therefore, it can be used to assess the level of individual innovation among nursing students and planning interventions in this field. In addition, nursing education researchers can also use this tool for descriptive and interventional studies in the field of individual innovation in nursing students.

## Background

Given the effective role of nursing students in promoting the nursing profession, they should learn to be innovative and create and use innovation to identify their educational needs and play an effective care role [1, 2].

All over the world, higher education administration programs, nursing curricula as well as nursing teachers seek to nurture nurses who are innovative, competent, and responsive to global needs [3]. In this regard, one of the primary necessities for fostering and nurturing innovation in nursing students is to study the level of individual innovation in them [4]. Individual innovation means the desire to search and find new approaches to problem solving using available technologies and applying these new approaches [5]. Having a suitable tool developed based on the culture and context of nursing student’s society is essential for evaluating the level of individual innovation in them.

Based on a review of available literature, so far no Persian scale has been designed to assess the level of individual innovation in nursing students. One of the available tools for investigating individual innovation is the individual innovativeness scale (IIS) by Hurt et al. (1977). This scale was designed to assess the level of individual innovation in students and teachers of the United States [6, 7]. The validity and reliability of this scale were assessed among Turkish nursing students [8] in 2010 and among Turkish nurses in 2013–2014, and the findings revealed that it was a reliable and valid scale in nursing [9]. IIS was also used in two separate studies in 2019 [1] and 2021 [3] to assess the level of individual innovation among nursing students in Turkey. So, the Hurt et al. (1977) tool was chosen for the following reasons:

- This scale assesses individual innovation in an educational context [7].
- The scale has good validity and reliability data in the US [7] and UK [10].
- The scale has good validity and reliability in other cultures/contexts (nursing community of Turkey) [8, 11], which has a cultural and social context close to the cultural and social context of Iran.

Since there is no tool in Persian to assess the level of individual innovation among Iranian nursing students and given the cultural differences between societies, the translation and cultural compatibility of this tool is essential. Therefore, this study was designed aiming at translation and psychometric analysis of IIS among Iranian nursing students.

## Methods

### Design

This cross-sectional study was conducted between September 2020 and June 2021 and designed to assess the validity of the IIS in the nursing students of School of Nursing and Midwifery of Shiraz University of Medical Sciences.

### Individual innovativeness scale (IIS)

IIS was designed to investigate the level of individual innovativeness of the college students and their teachers in the United States [7]. The premise of the developers of this scale is that innovation is normally distributed and unidimensional characteristic of people who compose a social system [10]. In order to extract items and form a pool of items, they had benefited from the literature review on the characteristics of the five innovation categories discussed by Rogers and Shoemaker (1971) [12]. IIS is a unidimensional scale (without subscales) comprising of 20 items. Twelve items are positive and 8 are negative. IIS is scored based on a 5-point Likert scale. The score range is between 14 and 94. To calculate the score, first the score of items 4, 6, 7, 10, 13, 15, 17, and 20 (step 1) and then the score of items 1, 2, 3, 5, 8, 9, 11, 12, 14, 16, 18, and 19 are added together (step 2). To obtain the total score, the following formula is used:

Innovativeness score = 42 + total score for step 2 - total score for step 1.

Individuals achieving a score above 68 are regarded as innovators. The reliability of IIS is 0.94 based on Nunnally’s technique (1967) [6, 7].

### Study participants

The minimum sample size required for exploratory factor analysis (EFA) is 3 to 10 samples per item [13]. Therefore, in this study, 7 individuals were included for each item. From among nursing students studying for the bachelor’s, master’s, or doctorate level, 140 individuals were selected by simple random sampling method if they wished to participate in the study. Samples were excluded from the study if they did not answer 5 items or more, which ultimately no individual was excluded.

According to studies, the minimum sample size proposed for estimating Cronbach’s alpha was 30 [14, 15], which in the present study was taken into account by considering a sample size of 140.

### Data collection

In this study, the demographic information collection form including gender, age, educational level and grade point average and the individual innovativeness scale of Hurt et al. (1977) were used to data collection.

This study was conducted in two phases, namely translation of IIS and psychometric analysis of IIS. The procedure and characteristics of each phase is described in the following.

### Phase 1: translation of IIS

The Forward-backward translation was conducted using the World Health Organization (WHO) model [16]. Forward translation from English to Persian was completed by two bilingual translators (Nursing professors who had full command of the English language at the native level and are familiar to the individual innovation concept). Then, the transcripts were combined by the authors, and the combined version was reviewed, edited and approved by a panel of experts in nursing, education and English language in one meeting. Backward translation from Persian to English was done by another translator (Who was expert in the field of English translation and was familiar with Iranian culture). Then, the first two translators compared the original version and the English translated version with each other and confirmed its conceptual similarity. Hence, the Persian version of IIS was confirmed [17].

### Phase 2: psychometric analysis of IIS

Psychometric properties studied at this phase included validity (face, content, and construct validity) and reliability (internal consistency and stability) [17].

#### Face validity

##### Qualitative face validity

The questionnaire was distributed among 15 people specializing in nursing and instrument development. Then, they were asked to judge the appropriateness, comprehensiveness, and relevance of the items.

##### Quantitative face validity

To check the face validity, they were asked to indicate the importance of each item through 5-point Likert scale (1 = not important at all, 5 = absolutely important). Accordingly, the impact score was calculated and items with impact score above 1.5 were preserved [13, 17].

#### Content validity

##### Qualitative content validity

IIS was provided to 15 experts in nursing and instrument development (13 individuals with PhD in nursing and 2 individuals with Master’s degree in nursing) and one expert in Persian language and literature. They were asked to evaluate items in terms of grammar, use of appropriate words, and wording.

##### Quantitative content validity

In order to check content validity ratio (CVR), the experts examined the necessity and usefulness of the items through 3-point Likert scale (1 = not necessary to 3 = necessary). Accordingly, CVR was calculated for each item. CVR above 0.49 was considered acceptable according to the Lawshe’s table. Then, the necessary revisions were made based on the experts’ opinions. In addition, to check content validity index (CVI), items were again provided to experts to evaluate their simplicity, relevance, and clarity based on 4-point Likert scale (1 = not related to 4 = completely related) [13, 17]. CVIs of each item and the whole questionnaire were then calculated. Items with CVI above 0.8 were retained [13, 17].

#### Construct validity

The construct validity was examined through exploratory factor analysis. Exploratory factor analysis was performed using varimax rotation method and taking into account eigenvalue above 1 and factor loading greater than 0.3. The sample size was considered suitable if Kaiser –Meyer-Olkin was higher than 0.5 [13].

#### Reliability

The reliability of IIS was assessed through studying internal consistency and item stability. Due to use of Likert scale, the internal consistency was examined by calculating Cronbach’s alpha, and Cronbach’s alpha above 0.7 was considered acceptable [13, 18].

To evaluate temporal stability of IIS, 30 under graduate nursing students (that were excluded from the study) were asked to complete the questionnaire again two weeks apart. Then, intraclass correlation coefficient (ICC) was calculated, and ICC value ≥ 0.8 was considered acceptable [19].

### Data analysis

Data analysis was performed using SPSS (version 25). Data were analyzed using descriptive (frequency/percentage, mean ± SD) and analytical (factor analysis rotation, correlation, Cronbach’s alpha coefficient and ICC) statistics while considering a significance level of 0.05.

## Results

The majority of participants in the study were female (72.14%), and studying for a bachelor’s degree in nursing (70%). The mean age of participants was 26.09 ± 6.61, and the mean IIS was 68.68 ± 10.37.(Table 1).

**Table 1:** Demographic characteristics and IIS of participants

Variable		N (%)	Mean (SD) of IIS
Gender	Male	39(27.86)	67.19(11.54)
	Female	101(72.14)	69.2(9.88)
Marital status	Single	100(71.42)	67.29(10.10)
	Married	40(28.58)	72.16(10.34)
Education level	Bachelor	98(70)	66.57 (9.95)
	Master of Science	21(15)	71.59(9.89)
	Doctorate	21(15)	75.68(9.33)

### Results of face validity evaluation

Fifteen experts in nursing and instrument development confirmed the appropriateness, comprehensiveness, and relevance of the items, and the impact score was above 1.5 for all items (Table 2).

**Table 2:** The item impact scores, CVR values, and CVI values of the Persian version of IIS

	Items	CVR	CVI	Impact score
1	My peers often ask me for advice or information.	0.86	0.8	4.96
2	I enjoy trying new ideas.	1	1	5
3	I seek out new ways to do things.	1	1	4.93
4	I am generally cautious about accepting new ideas.	1	0.93	4.8
5	I frequently improvise methods for solving a problem when an answer is not apparent.	0.86	0.93	3.55
6	I am suspicious of new inventions and new ways of thinking.	0.86	0.86	3.46
7	I rarely trust new ideas until I can see whether the vast majority of people around me accept them.	0.86	0.93	4.03
8	I feel that I am an influential member of my peer group.	0.73	0.86	3.75
9	I consider myself to be creative and original in my thinking and behavior.	0.86	0.93	4.34
10	I am aware that I am usually one of the last people in my group to accept something new.	1	0.93	4.41
11	I am an inventive kind of person.	0.6	0.86	2.78
12	I enjoy taking part in the leadership responsibilities of the group I belong to.	0.73	0.86	3.46
13	I am reluctant about adopting new ways of doing things until I see them working for people around me.	0.86	0.86	4.09
14	I find it stimulating to be original in my thinking and behavior.	0.86	1	4.86
15	I tend to feel that the old way of living and doing things is the best way.	1	0.93	4.48
16	I am challenged by ambiguities and unsolved problems.	0.86	1	5
17	I must see other people using new innovations before I will consider them.	0.6	0.80	3.75
18	I am receptive to new ideas.	1	1	4.66
19	I am challenged by unanswered questions.	0.73	0.93	4.41
20	I often find myself skeptical of new ideas.	0.73	0.86	3.52

#### Results of content validity evaluation

The qualitative content validity was revised and confirmed by 15 experts. Quantitative content validity was also confirmed by calculating CVR and CVI which were between 0.6-1 and 0.8-1, respectively. Moreover, S-CVI average was 0.91 (Table 2).

#### Results of construct validity evaluation

**Fig. 1:**
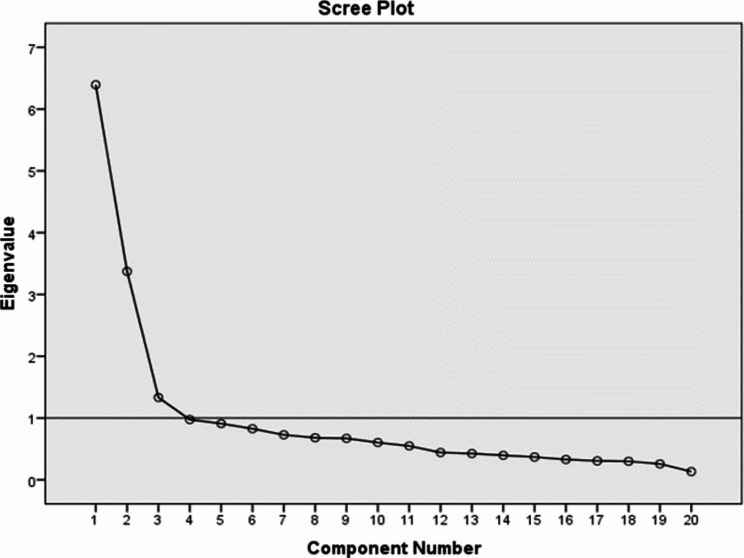
Screen plot to show the number of effective components to retain in the exploratory factor analysis (3 components = Resistance to change, Opinion leadership, Risk-taking)

Kaiser – Meyer‑Olkin value was 0.85, indicating the adequacy of the sample size. Based on factor analysis and scree plot, three factors were extracted with eigenvalue > 1, which cumulatively explained 55.49% of the changes in the questions (Fig. 1).

The percentage of variance explained for the first, second, and third subscale were 20.71, 19.98, and 14.96, respectively. The first subscale (resistance to change) included items 4, 6, 7, 10, 13, 15, 17, and 20, the second subscale (opinion leadership) included items 1, 2, 3, 5, 8, 9, 11, 12, and 19, and the third subscale (risk-taking) included only three items, including items 14, 16, and 18. The factor loadings ranged from 0.31 to 0.79 (Table 3).

**Table 3:** Factor loadings of IIS items

	Items	Resistance to change	Opinion leadership	Risk-taking
1	My peers often ask me for advice or information.	0.082	***0.494***	0.256
2	I enjoy trying new ideas.	0.204	***0.513***	0.491
3	I seek out new ways to do things.	0.195	***0.553***	0.495
4	I am generally cautious about accepting new ideas.	0.662	0.250	− 0.208
5	I frequently improvise methods for solving a problem when an answer is not apparent.	− 0.050	***0.687***	− 0.047
6	I am suspicious of new inventions and new ways of thinking.	***0.751***	0.074	0.119
7	I rarely trust new ideas until I can see whether the vast majority of people around me accept them.	***0.794***	0.057	0.103
8	I feel that I am an influential member of my peer group.	0.039	0.744	0.234
9	I consider myself to be creative and original in my thinking and behavior.	0.039	0.842	0.089
10	I am aware that I am usually one of the last people in my group to accept something new.	***0.668***	− 0.134	0.363
11	I am an inventive kind of person.	0.025	***0.808***	0.275
12	I enjoy taking part in the leadership responsibilities of the group I belong to.	0.224	***0.315***	0.258
13	I am reluctant about adopting new ways of doing things until I see them working for people around me.	**0.557**	0.080	0.463
14	I find it stimulating to be original in my thinking and behavior.	0.081	0.348	***0.672***
15	I tend to feel that the old way of living and doing things is the best way.	**0.650**	− 0.227	0.410
16	I am challenged by ambiguities and unsolved problems.	0.013	0.198	***0.678***
17	I must see other people using new innovations before I will consider them.	***0.693***	0.130	− 0.076
18	I am receptive to new ideas.	0.314	0.253	***0.685***
19	I am challenged by unanswered questions.	0.037	***0.523***	0.458
20	I often find myself skeptical of new ideas.	***0.774***	0.048	0.241
	Eigenvalue	4.14	3.96	2.99
	% variance	20.71	19.98	14.96
	Cumulative %	20.71	40.53	55.49

#### Results of reliability analysis

Findings from our analyses indicate the revised IIS for the Persian language produced scores with high levels of internal consistency (alpha = 0.88 for 20 items). In addition, with respect to stability analysis through test-retest, ICC was equal to 0.949 with a 95% confidence interval ranging from 0.894 to 0.976.

## Discussion

The aim of this study was translation and psychometric analysis of individual innovativeness scale among Iranian nursing students. Based on the results, the items of the questionnaire were appropriate to be used for measuring individual innovation in Iranian nursing students. In other words, according to the experts’ point of view, face and content validity of IIS was acceptable regarding CVI and CVR values. In Kemer’s study (2010) on Turkish nurses, the content validity of IIS was also confirmed (CVI = 0.91) [9]. The findings of Pallister’s study (1998) on psychometric properties of Hurt et al., (1977) scale, also yielded the appropriate discriminant validity of IIS [10].

In this study, construct validity was examined through exploratory factor analysis. EFA was used to extract latent factors from the newly translated scale and comparing it to the structure of the original scale to verify that the same factors with a similar organization of items within each factor are present [20]. Other cross-cultural adaptation studies have also used this method for evaluating construct validity [21]. In the study in which the original version of the Hurt et al.’s scale was developed, despite the fact that a two-dimensional structure emerged as a result of the factor analysis conducted, items were observed to accumulate in one dimension [7]. But in the present study, three dimensions of resistance to change (8 items), opinion leadership (9 items), and risk-taking (3 items) were obtained following exploratory factor analysis of the Persian version of IIS, which justified 55.49% of the changes in the questions. In Pallister’s psychometric research (1998) on 4 different consumer groups (retirement, life assurance, mortgage, and investment), a 5-dimensional scale was obtained considering all groups and a 4-dimensional scale was obtained considering each group alone [10]. In another study on Turkish nursing students, 4 dimensions of risk-taking, opinion leadership, openness to experience, and resistance to change were found, which explained 52.51% of the variance [8]. But in line with the present study, The Turkish version of IIS, which was psychometrically evaluated in the nurses also had three dimensions of risk-taking, opinion leadership, and resistance to change, which explained 49% of the total variance [9]. It maybe possible to say that this differences in the dimensions of the IIS in different studies is due to the conducting the study in different populations with different context and culture. However, in the studies conducted in Turkey, due to the proximity to the current study population, dimensions similar to the current study have been obtained.

The factor loading of the items ranged from 0.31 to 0.79, indicating the appropriateness and applicability of the questionnaire to assess individual innovation in nursing students. The factor loading of the main version of IIS was between 0.52 and 0.76 [7]. It was between 0.32 and 0.82 in Pallister et al.’s study (1998) [10], between 0.36 and 0.78 in the study done on Turkish nursing students [8], and between 0.49 and 0.75 in the study done on Turkish nurses [9].

The results of the present study suggested the appropriate internal consistency of IIS. Cronbach’s alpha value reported by Hurt et al.’s study (1977) [7] was also close to those obtained in the present study (C *α* = 0.89) and Pallister et al.’s study (1998) [10] (C *α* = 0.80). Cronbach’s alpha in the studies done on the Turkish students and nurses was 0.82 [2, 13]. In addition, the test-retest results also showed acceptable stability of the Persian version of IIS. Using test halving method, Hurt et al., (1977) reported a correlation of 0.92, indicating the evidence for stability of IIS [7]. Kılıçer (2010) also performed test-retest analysis on 61 nursing students two weeks apart and obtained a high and significance positive correlation between the two tests (p < 0.05, r = 0.87) [8]. Furthermore, the study of 74 Turkish nurses at 15-day interval showed a positive and significance correlation between the two phases of the test (r = 0.60, p = 0.000) [9]. Therefore, it seems that this questionnaire has a good reliability for being used to assess individual innovation in nursing students.

One of the limitations of the present study was the recruitment of students of one university. Therefore, larger studies using larger sample size and countrywide multicenter studies are recommended in this field. Furthermore, innovativeness is different between undergraduate and post-graduate students, but in the present study participants were selected from both groups. Also in qualitative evaluation of face and content validity the nature of expert’s opinion is subjective, therefore quantitative face and content validity were also investigated in this study. In addition to this, the construct validity has not been investigated by confirmatory factor analysis, so it is recommended to investigate the construct validity with this approach in future studies.

## Conclusion

Based on the results of this study, IIS is a valid and reliable tool for assessing the level of individual innovation in nursing students. The first step in developing students’ individual innovation is to examine their current status of innovation. Therefore, having a suitable tool in this field can be of great help to those involved in nursing education for investigating the level of individual innovation of nursing students and planning interventions in this field. In addition, nursing education researchers can also use this tool for descriptive and interventional studies in the field of individual innovation.

## Data Availability

The datasets analyzed during the current study are available from the corresponding author on reasonable request.

## List of abbreviations

CVI: Content Validity index
CVR: Content Validity Ratio
IIS: Individual Innovativeness Scale
